# High-throughput discovery of novel small-molecule inhibitors of acid Ceramidase

**DOI:** 10.1080/14756366.2022.2150183

**Published:** 2022-12-15

**Authors:** Mazen Aseeri, José Luis Abad, Antonio Delgado, Gemma Fabriàs, Gemma Triola, Josefina Casas

**Affiliations:** a Department of Biological Chemistry, Institute for Advanced Chemistry of Catalonia (IQAC-CSIC), Barcelona, Spain; b Department of Pharmacology, Toxicology and Medicinal Chemistry, Unit of Pharmaceutical Chemistry (Associated Unit to CSIC), Faculty of Pharmacy and Food Sciences, University of Barcelona, Barcelona, Spain; c Liver and Digestive Diseases Networking Biomedical Research Centre (CIBEREHD), ISCIII, Madrid, Spain

**Keywords:** Sphingolipids, ceramide, acid ceramidase, inhibitor

## Abstract

Ceramide has a key role in the regulation of cellular senescence and apoptosis. As Ceramide levels are lowered by the action of acid ceramidase (AC), abnormally expressed in various cancers, the identification of AC inhibitors has attracted increasing interest. However, this finding has been mainly hampered by the lack of formats suitable for the screening of large libraries. We have overcome this drawback by adapting a fluorogenic assay to a 384-well plate format. The performance of this optimised platform has been proven by the screening a library of 4100 compounds. Our results show that the miniaturised platform is well suited for screening purposes and it led to the identification of several hits, that belong to different chemical classes and display potency ranges of 2–25 µM. The inhibitors also show selectivity over neutral ceramidase and retain activity in cells and can therefore serve as a basis for further chemical optimisation.

## Introduction

Sphingolipids (SLs) are a major class of cellular lipids. Besides playing a structural role in cellular membranes, several members of the SLs family are also involved in the regulation of a variety of cellular processes. The metabolic hub in SLs biosynthesis and catabolism is ceramide, a bioactive lipid intimately involved in the regulation of stress response^1^, inflammation^2^, apoptosis^3^ and cancer cell death^4^.

In the recent years, there is more and more evidence that maintaining a tight regulation of ceramide levels is key to cells and strongly contributes to cell fate decisions. Moreover, altered ceramide levels are a hallmark in the manifestation of several pathological processes such as Alzheimer disease^5^, metabolic disorders^6^ or cancer^7^, in which lower levels of this lipid are inversely correlated with the degree of malignant progression^8^. Consequently, tremendous efforts have been devoted to identifying small molecules targeting the enzymes involved in ceramide biosynthesis and degradation.

Ceramide can be generated *de novo* from serine and palmitate, by the degradation of sphingomyelin catalysed by sphingomyelinases and by the acylation of sphingosine in the salvage pathway. Ceramide degradation is in turn mediated by the actions of different ceramidases that are distinguished by the pH required for optimal activity, i.e. acid ceramidase (AC, ASAH1), neutral ceramidase (NC, ASAH2) and alkaline ceramidases 1, 2 and 3 (ACER1, ACER2 and ACER3)^9^. Different functions and cellular roles, probably defined by their intracellular localisation and substrate specificity, have been suggested for these ceramidases. Hence, NC overexpression has been related to colon carcinogenesis^10^, whereas ACER3 has been reported to contribute to hepatocellular carcinoma^11^ and to acute myeloid leukaemia pathogenesis^12^.

AC is one of the better-characterized ceramidases and its role in cancer initiation and progression has been largely studied. Abnormally elevated AC expression has been reported in various type of cancer including prostate cancer^13^, colon adenocarcinoma^14^, head and neck cancer^15^, glioblastoma^16^ and melanoma^17^. Moreover, whereas AC overexpression renders the cells more resistant to chemo and radiotherapy^18^, inhibition of the enzyme sensitises the cell to treatment^19^, thereby suggesting a role of AC in drug resistance associated to therapy. As a result, AC inhibition has emerged as an attractive target to improve the efficacy and lower the resistance to cancer treatments, and the identification of novel and selective AC inhibitors has gained increasing interest. Tremendous efforts have been done during the last two decades to develop AC modulators. However, most of the reported inhibitors are structurally related to ceramide, which has a negative impact on their selectivity, potency and drug-like properties^20^. Thus, the discovery of ceramide-unrelated hits would be highly desirable. Some potent and structurally unrelated inhibitors of AC have already been described. Representative examples of this class of compounds are carmofur, identified after the screening of a commercial library using a LC/MS-based assay^14^, and the related dioxypyrimidine and benzoxazolone carboxamides^21–23^. Despite these relevant examples, there is still a great need for the identification of novel molecules that can expand the toolbox for AC inhibitors.

One of the best alternatives to identify structurally diverse inhibitors is through the screening of large compound libraries. However, this approach requires a powerful, robust and cost-effective HTS assay, allowing the rapid and reliable testing of large number of compounds. Recently, we described a flow-cytometry-based assay that uses a deoxyceramide analog to monitor AC activity in intact cells. This assay could be potentially useful in the future for screening purposes, but it would require proper optimisation and the use of high-throughput flow cytometry platforms^24^. Herein, we report a fluorescence-based AC assay that has been adapted to a 384-well format. Once validated, it has been employed to evaluate a 4100 compound library leading to the identification of novel compound classes targeting AC activity. Remarkably, the identified inhibitors also show selectivity over NC and retain activity in cellular studies.

## Results and discussion

**Scheme 1.**
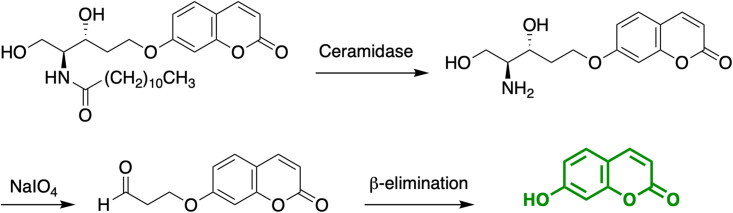
Enzymochemical transformation of RBM14-C12 into fluorescent umbelliferone.

**Figure 1.**
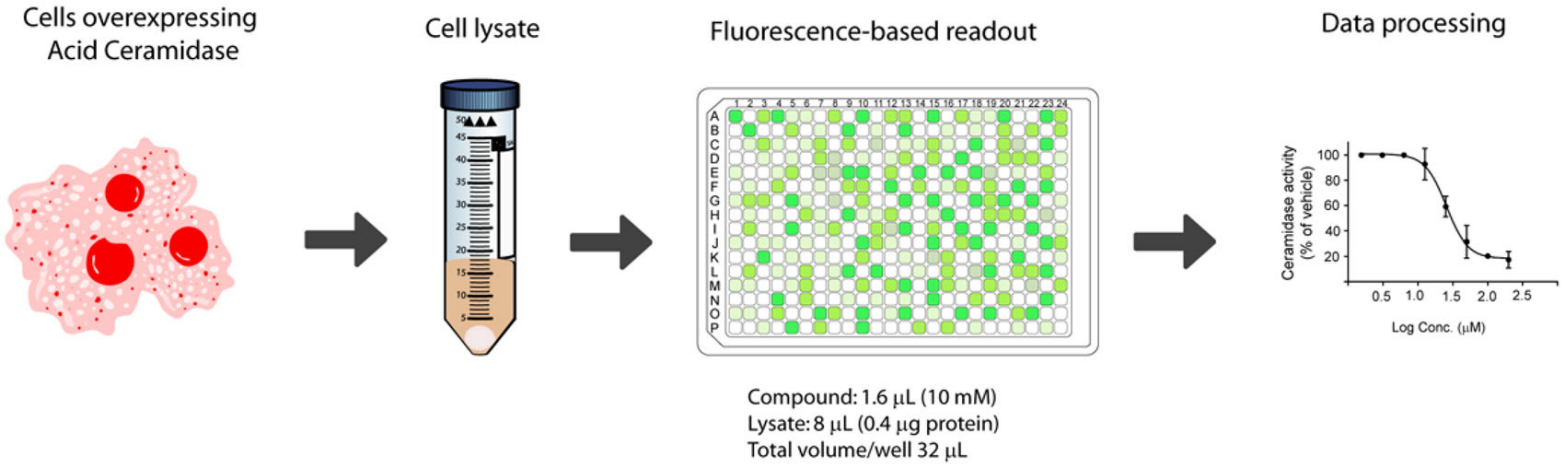
Workflow for acid ceramidase activity screening in the 384-well HTS format.

The screening for specific and potent AC inhibitors requires the availability of a high-throughput screening assay (HTS) capable of examining relatively large number of compounds simultaneously. A fluorescent-based assay in a 96-well format was previously set up in our group and applied to measure acid ceramidase activity using cell lysates and intact cells as a source of enzyme. The assay is based on the coumarinic substrate RBM14-C12^25^, that shows a high affinity and specificity for AC over neutral and alkaline ceramidases. Briefly, hydrolysis of the amide bond of RBM14-C12 by AC yields an aminodiol that can be then oxidised upon treatment with sodium periodate. The resulting aldehyde undergoes a β-elimination reaction to release the fluorescent product umbelliferone (Scheme 1). The assay has been largely used by our group and others to identify novel ceramidase inhibitors^21^^,^^26^^,^^27^. However, assays with increased capacity are required to allow the rapid high-throughput screening (HTS) of large chemical libraries, thereby accelerating the drug discovery process as well as reducing the associated costs. Remarkably, recent advances have been performed in this area of research. Thus, a HTS screening assay for the identification of neutral ceramidase inhibitors was recently established by Spicer et al. using an analogous substrate RBM14-C16, developed in our group^28^. Moreover, Granier et al. synthesised a suite of doubly fluorophore-modified ceramides as turn-on probes for the direct FRET-based analysis of ceramidase activity in real-time. Although ACER3 was able to hydrolyse one of the probes, no synthetic substrates were hydrolysed by AC^29^. Therefore, although the Granier’s method has the advantage of monitoring ceramidase activity in real-time, it cannot be applied to AC until suitable FRET ceramide substrates are discovered for this enzyme. Herein, we report the miniaturisation of an AC assay to a 384-well format and we employ it for the identification of new acid ceramidase inhibitors. The robustness and assay performance of the miniaturised assay were validated and assessed by the screening of a 4100 compound library, leading to the identification of novel AC inhibitors (Figure 1).

### Optimisation and miniaturisation of the AC HTS assay

**Figure 2.**
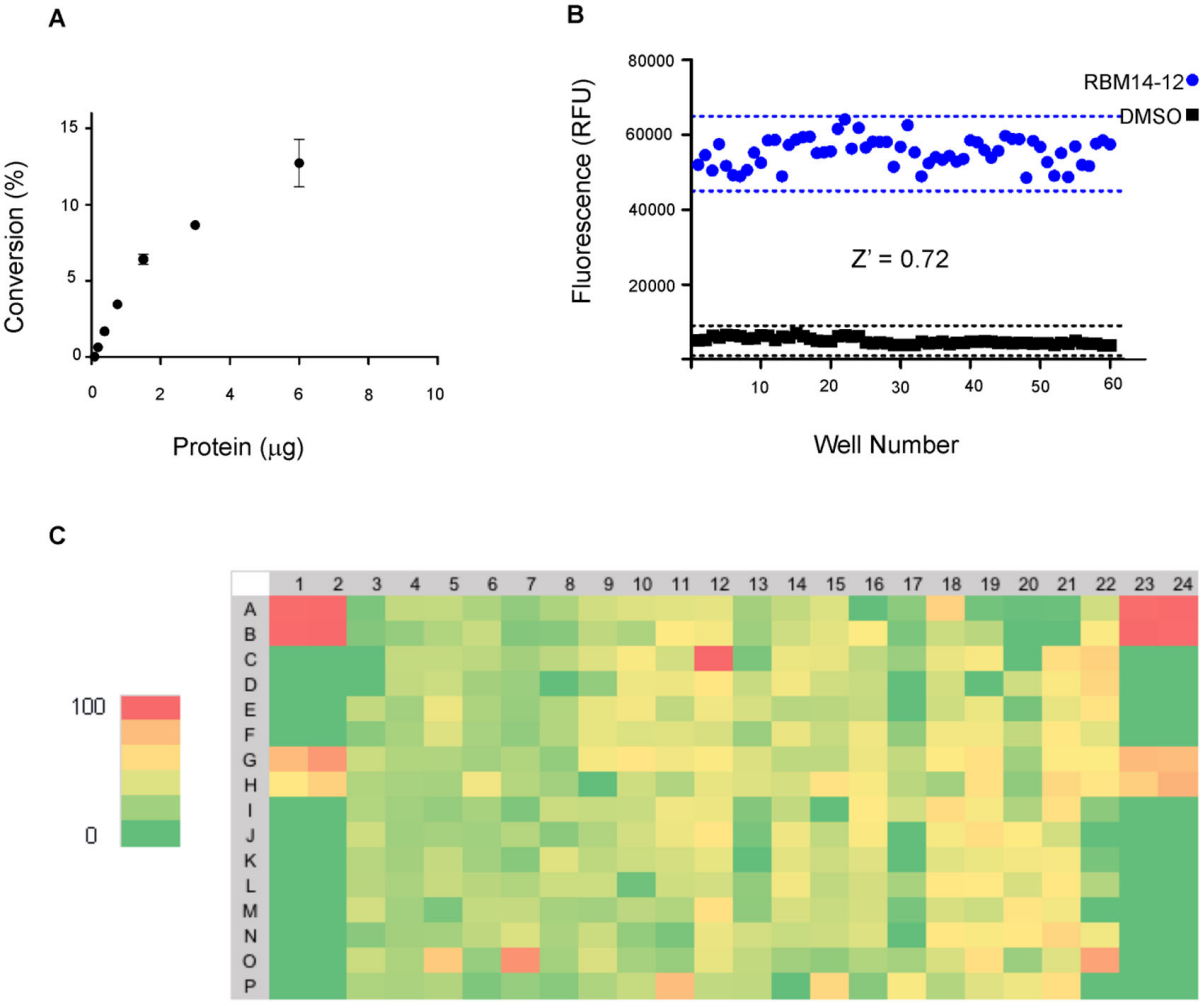
(A) Percentage of substrate conversion obtained using different amounts of AC (measured as μg of total protein). (B) Determination of the *Z*’-factor (*Z*’) of the AC assay in the 384-well plate format. Solid black squares and solid blue circles represent negative (without protein) and positive reaction, respectively. (C) Representative results for one 384-well plate. Each plate contained 8 negative controls, 16 positive controls and 8 samples with the known AC inhibitor SOCLAC. A colour gradient heat map, with red to green colours indicating high to low percentage of inhibition has been applied to the well values.

To start with, the final reaction volume was adapted from 100 µL (96-well-format) to 32 µL (384 well-format) with a final concentration of substrate of 20 µM. Next, protein concentration was adjusted using cell lysates of AC-overexpressing A375 melanoma cells. Ideally, the optimal amount of protein is the one that ensures reaction linearity over a period of time together with less than 10% of substrate depletion, in order to assume that the enzyme operates at steady-state conditions. Thus, different amounts of cell lysates were mixed with the substrate and product formation was measured over 1 h. Figure 2(A) displays the reaction progress curve for the hydrolysis of RBM14-C12 by AC obtained at a range of protein concentrations. Finally, an amount of 0.4 µg of protein was chosen, as higher amounts of proteins were not in the linear portion whereas lower amounts would compromise the signal window. The optimal incubation time of the reaction mixture was next explored. Although a higher signal was witnessed at 2 h reaction time, a final reaction time of 1 h was chosen since it exhibited an excellent signal window. As a first validation step, we performed a control run consisting of 60 wells representing AC activity and 60 wells representing the signal in absence of the enzyme. Figure 2(B) depicts the results and reveals excellent separation between high and low control wells resulting in a signal-to-background ratio >8 and a *Z ’* factor of 0.72. The calculated coefficients of variation (CV) for both the high and low controls were 6.08% and 17.1% respectively. These data demonstrated that the assay was very robust, stable and possessed minimal well-to-well variability and therefore it is appropriate for HTS to identify AC inhibitors.

### Screening of a compound library

After having successfully established the optimal reaction conditions in a 384-well format, we employed this newly miniaturised AC assay in a pilot screen against a library of 4100 compounds. The screen was carried out at a single dose of 20 µM for each library compound in 6% DMSO (v/v) and in duplicate. The acid ceramidase inhibitor SOCLAC was used as a pharmacological control for the assay^26^. The Z’-factor per plate were consistent with those obtained during the initial validation. A total of 116 compounds of the 4100 screened at 20 µM met the active criterion, based on the hit cut-off at % inhibition >40, in the single-point primary screen, what resulted in a hit rate of 2.8%. An example hit map from one screening plate is shown (Figure 2(C)). The inhibitory activity of these hits was then validated in an additional assay performed at a single-point concentration in triplicates.

**Figure 3.**
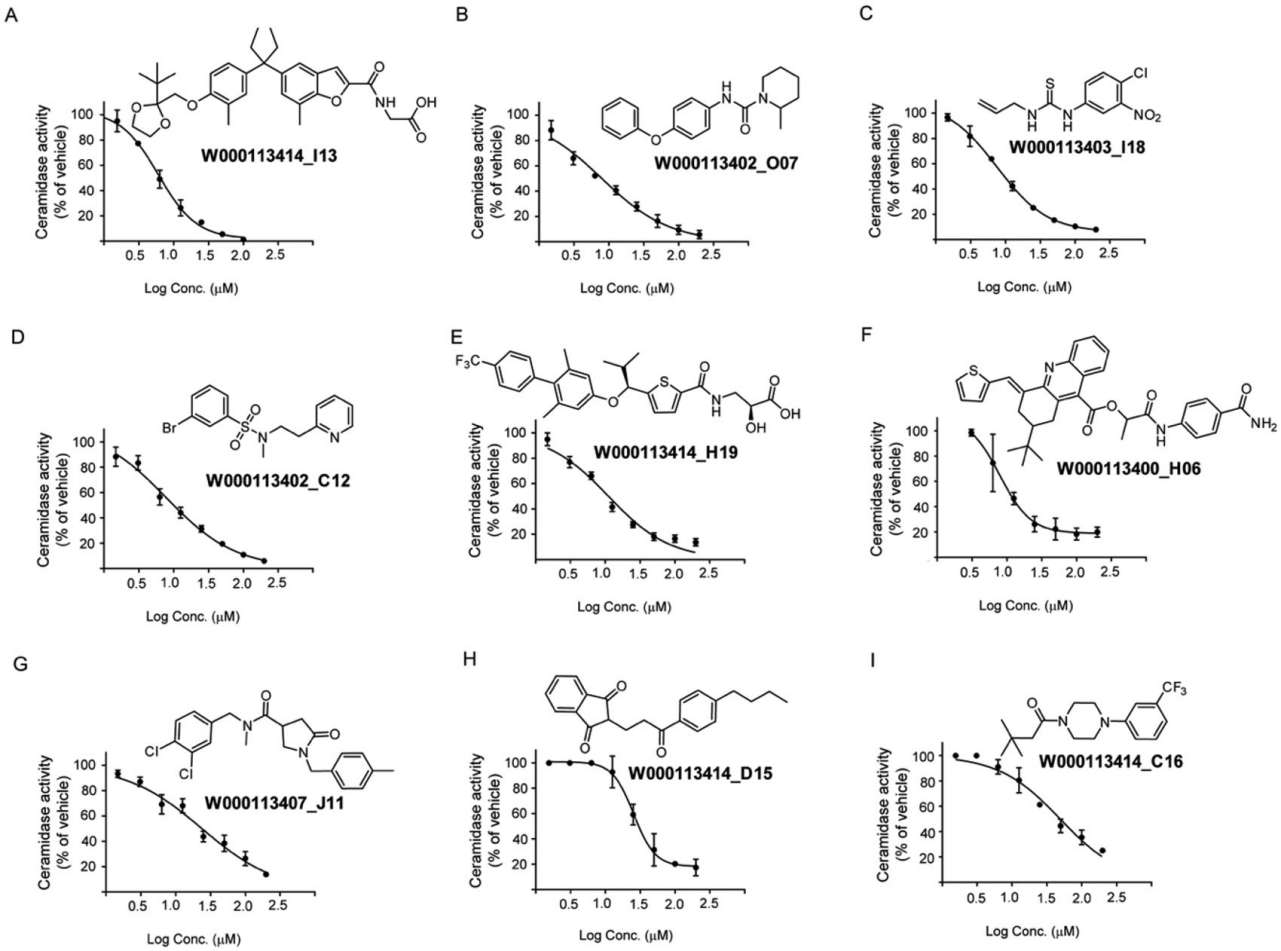
Structure of the identified inhibitors and their concentration-response curves. Active compounds were dose-tritiated to establish the corresponding IC_50_ values (*N* = 3 well per replicate point, errors bars are shown).

To confirm the results from the primary screening, top hits were cherry-picked and tested in a concentration-dependent response assay with triplicates per sample. Thus, 101 compounds were then subjected to 8-points two-fold dilution series starting at a maximum concentration of 200 micromolar. Among them, nine exhibited dose-dependent inhibition of AC with IC_50_ values in the range of 6 and 49 µM (Table 1, Figure 3), whereas 92 compounds showed a higher IC_50_ value or did not yield a concentration-dependent inhibition (structures not disclosed).

**Table 1.** The hit molecules identified through the primary assay performed at a single-point concentration of 20 μM were dose-titrated to establish their IC_50_ values (95% confidence interval).

Unique ID	% Inhibition	IC_50_ (µM )
W000113414_I13	67.39	6.6 (9.5–12.9)
W000113402_O07	53.75	7.9 (6.9–9.0)
W000113403_I18	63.38	10.6 (9.5–11.7)
W000113402_C12	75.46	10.6 (9.3–12.0)
W000113414_H19	61.39	11.1 (9.5–12.9)
W000113400_H06	41.71	15.8 (11.1–22.8)
W000113407_J11	59.17	23.9 (20.1–28.4)
W000113414_D15	60.38	36.7 (30.2–45.5)
W000113414_C16	58.95	48.9 (41.9–57.5)

Values shown are means of three replicates experiments.

### Inhibition of neutral ceramidase

As mentioned above, hydrolysis of ceramides occurs by the action of ceramidases which are encoded by five known genes and are distinguished by the pH required for optimal activity^9^. A common problem with AC inhibitors, especially those one with a scaffold related to the structure of ceramide, is the lack of selectivity over other ceramidases. Thus, to discard a selectivity issue, the activity of nine detected hits against neutral ceramidase was next explored at 50 µM. Activity over NC was tested using recombinant human NC (rhNC) and a specific NC substrate bearing a nervonic acid amide (RBM14-C24:1)^30^. Remarkably, none of the molecules elicited a significant inhibitory activity, thereby confirming the selective inhibition of AC over NC (Supplemental Table 1).

### Cellular inhibition of acid ceramidase

**Figure 4.**
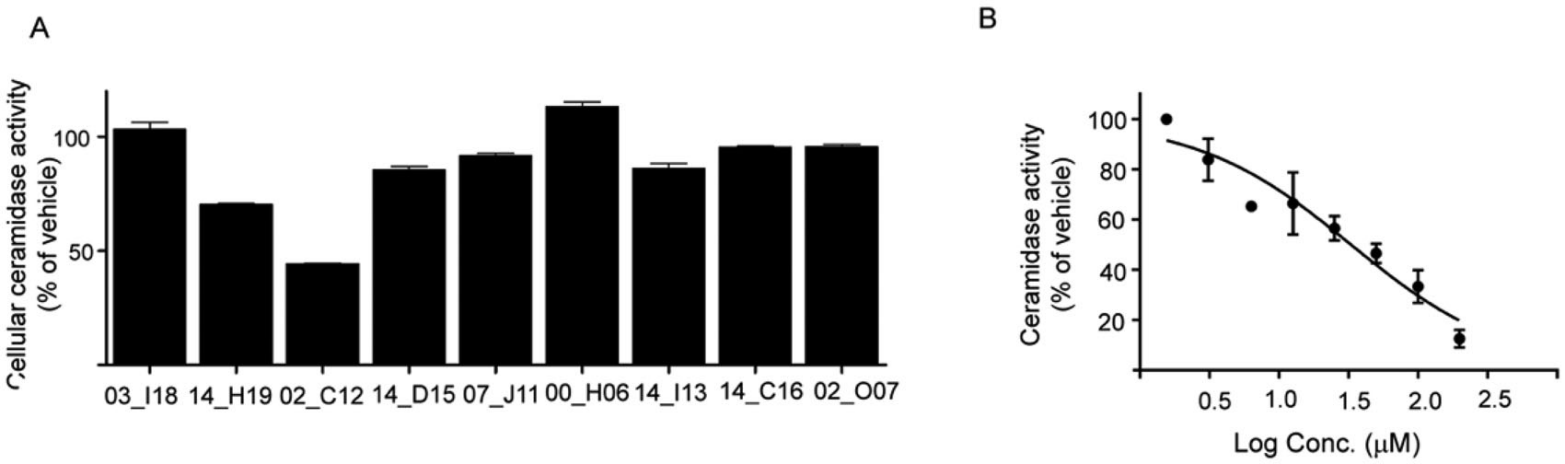
(A) Cellular validation of hit compounds on acid ceramidase. The hit molecules identified during the primary validation were investigated using a cellular assay. Values shown are mean of three replicates and results are expressed as percentage of activity compared to vehicle control. Compounds are identified with the last 5 letters/numbers of their unique code (B) Concentration-response curve of the most potent hit molecule W000113402_C12 in a cellular assay.

Cell-based assays allow evaluating biological activity in a more physiologically relevant system that also considers additional factors that might have a positive impact on inhibitory potency such as permeabilization through cellular membranes, metabolisation or concentration by intracellular compartmentalisation. Thus, the activity of the nine selected hits was next examined in intact cells using AC overexpressing A375 cells and the fluorogenic substrate RBM14-C12. First, compounds were tested in a primary screening assay. Measurements were performed in triplicated at a single-point concentration of 20 µM. The results showed that only two of the 9 tested compounds exhibited a significant inhibition of AC activity at this concentration: W000113402_C12 with a 53% inhibition and W000113414_H19 with a weaker 32% inhibition, whereas slight effects were observed for the other hits (Figure 4(A)). Dose-dependent inhibition could be also confirmed for W000113402_C12 in the cell-based assay displaying an IC_50_ of value of 32 µM (24.3–44.7) (Figure 4(B)).

## Summary and conclusions

HTS is still the primary hit-finding strategy both in academia and industry. However, the identification of novel AC inhibitors has been hampered by the unavailability of appropriate screening platforms. Herein, we report a robust and cost-effective assay for the determination of AC activity that enables the rapid profile of large compound libraries. The screening platform has been employed to evaluate a 4100 compound library leading to the identification of 9 novel compound classes targeting AC activity with low micromolar IC_50_. Dose-dependent inhibition was confirmed for the primary hits identified in the screening campaign and now they can serve as a basis for hit-to-lead optimisation through chemical modifications, thereby opening new venues in the field of AC inhibition. Moreover, the reported technique can be considered an attractive drug-screening platform with a great potential to identify novel AC modulators. To further validate hits obtained in HTS campaigns of large libraries, an orthogonal assay using a different detection method (e.g. C12-Ceramide Bodipy and HPLC-based detection of substrate and reaction product) could be applied to discard potential interference of compounds on the fluorescence-based assay^31^. As several diseases are linked to altered AC activity, novel compounds modulating its activity should allow progress in drug discovery and expand our knowledge in the essential role of this enzyme.

## Materials and methods

### Compound library

A library containing 4100 compounds was obtained from Eli Lilly. Compounds were distributed in triplicates in 384-well microtiter plates at a 10 mM concentration in DMSO (0.4 µL). Compounds were identified with a unique code. Plates were stored at −20 °C until use. Immediately prior to use, plates were withdrawn from −20 °C storage, thawed an ambient temperature and centrifuged.

### Cell culture

The A375 cell line stably overexpressing *ASAH1* under the control of a tetracycline/doxycycline-responsive promoter was kindly provided by Dr. Carmen Bedia and Prof. Thierry Levade^17^. The antibiotic selection of this cell line was performed with blasticidin (3 µg/mL) and hygromycin B (250 µg/mL). Ectopic expression of AC was induced with doxycycline at 1 µg/mL for 24 h before use. Cells were suspended in the appropriate volume of a 0.25 M saccharose solution with the proteases inhibitors aprotinin (1 mg/mL), leupeptin (1 mg/mmL) and PMSF (100 mM). The suspension was submitted to three cycles of a 5 s sonication (probe) at 10 watts/5 s resting on ice. The cell lysate was centrifuged at 600 g for 5 min. The supernatant was collected and protein concentration was determined with BSA as a standard using the bicinchoninic acid (BCA) protein determination kit (Thermo Scientific) according to the manufacturer’s instructions.

### Acid ceramidase HTS assay

A previously described 96-well plate assay^25^ was miniaturised into a 384-well plate format with a final reaction volume of 32 µL. Plated compounds were diluted with a mixture of DMSO/H_2_O (1.6 µL/18 µL), and after centrifugation, 3.2 µL were dispensed into a new 384-well plate, so that the final concentration of the compound in the final reaction volume of 32 µL was 20 µM and the DMSO content of the assay was 1%. Next, 20.8 µL of a substrate solution of RBM14-C12 in sodium acetate buffer (25 mM, pH 4.5) was added for a final concentration of 20 µM, followed by the addition of 8 µL of a 0.25 M sucrose solution of cell lysates from AC-overexpressing A375 melanoma cells containing 0.4 µg of protein The reaction was terminated after 60 min incubation at 37 °C by adding 8 µL of methanol. Oxidation was performed by treatment with 32 µL of a [2.5 mg/mL] solution of NaIO4 in 100 mM glycine-NaOH buffer (pH 10.6). The plates were incubated at 37 °C in the dark for another 1 h. Finally, 32 µL of 100 mM glycine-NaOH buffer (pH 10.6) were added and fluorescence was measured spectrophotometrically at excitation and emission wavelength of 355 and 460 nm, respectively. Blank reactions contained the same constituents as the test reactions except the cell lysates.

### Neutral ceramidase assay

The NC assay was performed in 96-well plates at a final volume of 100 µL/well. Reaction buffers was 25 mM phosphate buffer 150 mM NaCl 1% (NaChol) pH 7.4. The reaction mixtures contained 25 µL/well of protein (5 ng recombinant NC R&D Systems, >95% pure), 70 µL/well of substrate (prepared from 4 mM stock solutions in ethanol) and 5 µL/well of inhibitor (prepared from 1 mM stock solutions in DMSO/H_2_O). Reaction mixtures were incubated at 37 °C for 1 h and reactions were stopped with 25 µL/well of MeOH followed by 100 µL/well of NaIO4 (2.5 mg/mL in 100 mM glycine-NaOH buffer, pH 10.6). After incubation at 37 °C for 1 h in the dark, 100 µL/well of 100 mM glycine-NaOH buffer (pH 10.6) was added and fluorescence was measured spectrophotometrically at excitation and emission wavelenght of 355 and 460 nm, respectively. The same reaction mixtures without enzymes were used as blanks.

### Fluorogenic ceramidase activity assay in intact cells

To determine activity in intact cells, 2 × 10^4^ cells/well were seeded in 96-well plates 24 h prior to the assay and maintained at 37 °C and 5% CO_2_. Overexpression of AC was induced with doxycycline at 1 µg/mL for 24 h. Medium was replaced by 100 µL of fresh medium (DMEM 10% FBS) containing 20 µM of the substrate and different concentrations of the indicated test compounds. Both substrates and test compounds were added simultaneously to the cell culture. The plate was incubated for 3 h at 37 °C in 5% CO2. The reaction was stopped with 25 µL/well of MeOH and then 100 µL/well of NaIO_4_ (2.5 mg/mL in glycine-NaOH buffer, pH 10.6) were added. After incubation at 37 °C for 1 h in the dark, 100 µL/well of 100 mM glycine-NaOH buffer (pH 10.6) were added and fluorescence was measured spectrophotometrically at excitation and emission wavelengths of 355 and 460 nm, respectively. The same reaction mixtures without cells were used as blanks.

## Supplementary Material

Supplemental Material
