# Supplementary material for: High-throughput discovery of novel small-molecule inhibitors of acid Ceramidase
Source: J Enzyme Inhib Med Chem. 2022 Dec 15;38(1):343–8. doi: 10.1080/14756366.2022.2150183 (PMC9762759; doi:10.1080/14756366.2022.2150183)
Supplement: Supplemental Material [file IENZ_A_2150183_SM6524.pdf]

Supplementary Table

| Unique ID | % Inhibition Neutral Ceramidase |
|---|---|
| **W000113414_I13** | 22.9 |
| **W000113402_O07** | 8.5 |
| **W000113403_I18** | 23.4 |
| **W000113402_C12** | -5.9 |
| **W000113414_H19** | -8.9 |
| **W000113400_H06** | 35.6 |
| **W000113407_J11** | 20.8 |
| **W000113414_D15** | -1.3 |
| **W000113414_C16** | -4.1 |

Sup Table 1. The hit molecules in the acid ceramidase assay were investigated in a neutral ceramidase assay performed at a single-point concentration of 50 μM. Values shown are means of three replicates experiments.

Information Classification: General
